# Topological analysis of a haloacid permease of a *Burkholderi*a sp. bacterium with a PhoA-LacZ reporter

**DOI:** 10.1186/1471-2180-9-233

**Published:** 2009-10-31

**Authors:** Yuk Man Tse, Manda Yu, Jimmy SH Tsang

**Affiliations:** 1 Molecular Microbiology Laboratory, School of Biological Sciences, The University of Hong Kong, Pokfulam Road, Hong Kong

## Abstract

**Background:**

2-Haloacids can be found in the natural environment as degradative products of natural and synthetic halogenated compounds. They can also be generated by disinfection of water and have been shown to be mutagenic and to inhibit glyceraldehyde-3-phosphate dehydrogenase activity. We have recently identified a novel haloacid permease Deh4p from a bromoacetate-degrading bacterium *Burkholderia *sp. MBA4. Comparative analyses suggested that Deh4p is a member of the Major Facilitator Superfamily (MFS), which includes thousands of membrane transporter proteins. Members of the MFS usually possess twelve putative transmembrane segments (TMS). Deh4p was predicted to have twelve TMS. In this study we characterized the topology of Deh4p with a PhoA-LacZ dual reporters system.

**Results:**

Thirty-six Deh4p-reporter recombinants were constructed and expressed in *E. coli*. Both PhoA and LacZ activities were determined in these cells. Strength indices were calculated to determine the locations of the reporters. The results mainly agree with the predicted model. However, two of the TMS were not verified. This lack of confirmation of the TMS, using a reporter, has been reported previously. Further comparative analysis of Deh4p has assigned it to the Metabolite:H^+ ^Symporter (MHS) 2.A.1.6 family with twelve TMS. Deh4p exhibits many common features of the MHS family proteins. Deh4p is apparently a member of the MFS but with some atypical features.

**Conclusion:**

The PhoA-LacZ reporter system is convenient for analysis of the topology of membrane proteins. However, due to the limitation of the biological system, verification of some of the TMS of the protein was not successful. The present study also makes use of bioinformatic analysis to verify that the haloacid permease Deh4p of *Burkholderia *sp. MBA4 is a MFS protein but with atypical features.

## Background

Haloacids are metabolic products of naturally occurring compounds [1-3] and are also disinfection by-products of sewage and water [4,5]. It has been shown that some haloacids are toxic and mutagenic [6,7]. Microorganisms capable of degrading these haloacids can be found in the natural environment. One of these, a soil-borne *Burkholderia *sp., MBA4, has been isolated for its ability to grow on monobromoacetate (MBA) [8]. This bacterium produces a haloacid dehalogenase that allows the cell to grow on MBA. Since MBA is a more potent mutagen than ethylmethane sulfonate [9] one would not expect an uptake mechanism for this kind of compound. We have, however, identified a haloacids-transporter protein gene downstream of the dehalogenase gene. This haloacid permease, Deh4p, was expressed, together with the dehalogenase, to enhance the uptake of haloacetates [10]. The gene encoding for Deh4p has been cloned and expressed in *E. coli *which facilitated the specific uptake of haloacetates [11]. Deh4p is 552 residues long and has a putative molecular weight of 59,414 and an isoelectric point of 9.14.

With the blooming of the sequencing data and the development of bioinformatics, software that predicts the structure of a protein has become more and more readily available [12-21]. Topology prediction programs that use different algorithms are easily accessible from the Internet and their predictions are becoming more and more accurate. Comparative analysis of the primary structure of Deh4p with proteins in the Pfam database [22] has designated it as a member of the Major Facilitator Superfamily [23] (MFS, TC 2.A.1). MFS is a major class of membrane transporter with more than a thousand known proteins [24]. It is also described as the uniporter-symporter-antiporter family. Although there are many members in this family, only four of them have well defined structure or topology. These proteins are EmrD [25], LacY [26] and GlpT [27], all from *Escherichia coli *and OxlT from *Oxalobacter formigenes *[28,29]. They have been shown to possess twelve transmembrane segments (TMS) with a 2-fold symmetry roughly dividing the first and the second 6-TMS. The termini of these proteins were found to reside within the cytoplasm. Though MFS transporters with 14 and 24 TMS are known [30,31], they are relatively few in number [32]. Hence the presence of twelve TMS was believed to be the standard characteristic of the MFS proteins.

Notwithstanding the abundance and improved accuracy of those computer analysis methods, experimental determination is still necessary. The use of reporter fusion analyses is by far the most convenient method and the use of dual-reporters is no doubt a better choice than the use of a single indicator [33,34]. Here we report the experimental determination of the topology of Deh4p using a PhoA-LacZ dual-reporters system [33] and the verification using a comparative approach.

## Results

### Hydropathy analysis of Deh4p

**Figure 1:**
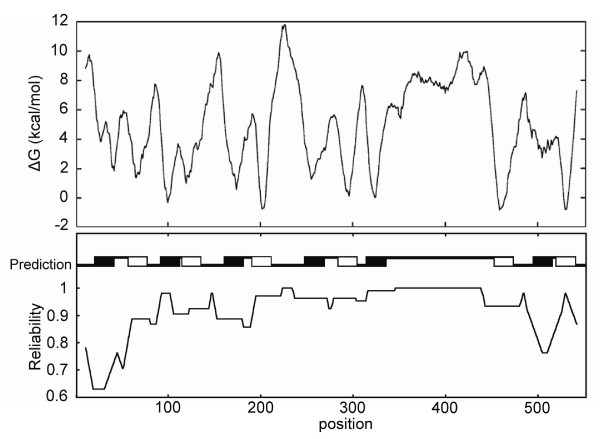
**A hydrophobicity plot of Deh4p**. A hydrophobicity plot based on the ΔGpred method [37] was produced by the TOPCONS server (topcons.cbr.su.se) [62]. The predicted transmembrane helices are indicated by black (helix from N_in _to C_out_) and white (helix from N_out _to C_in_) boxes, respectively. The reliabilities of the helices are also indicated.

Computational analysis of Deh4p has categorized it as a MFS protein. This classification was based on the following grounds. First, Pfam [22] analysis (accessed on 29 May, 2009) indicated that Deh4p is a member of the clan MFS and has a signature of PF00083 sugar (and other) transporter family. The signature, [LIVMF]-X-G- [LIVMFA]-{V}-X-G-{KP}-X(7)- [LIFY]-X(2)- [EQ]-X(6)- [RK] is found between residues 130 and 155 of Deh4p [10]. Second, the length of Deh4p, 552 residues, is within the known range of 400 to 600 for MFS [24] and third, it was predicted to have twelve TMS, typical for MFS, by many topology prediction programs such as OCTOPUS [20], TMpro [35], SOSUI [14] and PHDHTM [18]. The monochloroacetate uptake ability of Deh4p was inhibited in the presence of a proton motive force inhibitor, carbonyl cyanide 3-chlorophenyl hydrazone (Yu, unpublished result). This suggested that Deh4p is most likely a symporter or antiporter. When the topology of Deh4p was predicted using TMHMM [36] and SOSUI [14], the models were different from a typical MFS symmetrical arrangement. Deh4p has a long periplasmic loop, stretching from residues 337 to 454, near the C-terminal. Fig. 1 shows a hydrophobicity plot of Deh4p using ΔGpred algorithm [37]. The prediction showed that there were twelve TMS with the N- and the C-termini located in the cytoplasm. All except TMS 1 and 11 have reliability values of more than 0.75 and the fifth periplasmic loop has a value of 1. These suggested that the prediction was reasonably good and Deh4p is likely to be a MFS protein.

### Topological analysis using Deh4p-PhoA-LacZ fusions

**Figure 2:**
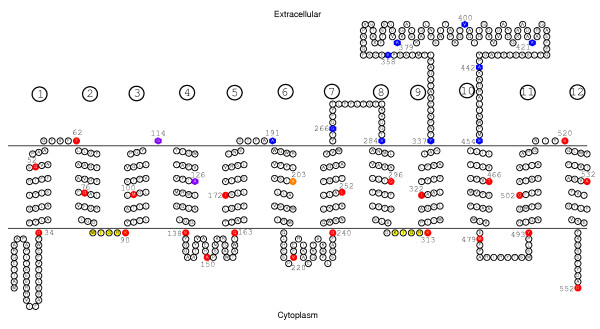
**A predicted topology of Deh4p**. A topological model of Deh4p derived from the SOSUI prediction (bp.nuap.nagoya-u.ac.jp/sosui). The relative locations of the fusion reporters are indicated by numbers and colored residues. Qualitative dual-reporters activities are shown as red-colored circles (the LacZ activity was at least 3-fold higher than the PhoA activity), blue-colored hexagons (the PhoA activity was at least 3-fold higher than the LacZ activity), orange-colored circle (the LacZ activity was higher than the PhoA activity but less than 3-fold), and purple-colored hexagons (higher PhoA than LacZ activity but less than 3-fold). The twelve putative TMS are also indicated as numbers in circles. The conserved MFS signature motif of [RK]XGR [RK] is highlighted in yellow.

Although most of the predicted models of Deh4p exhibited twelve TMS it is necessary to validate these predictions experimentally. The use of reporter fusions technique is a commonly used practice. In this study we utilized a dual-reporters system. Bacterial alkaline phosphatase (PhoA) is an enzyme that functions only in the periplasmic space [38] while β-galactosidase (LacZ) is an enzyme that works only in the cytoplasm [39]. The use of these PhoA-LacZ dual-reporters in topology studies gives more reliable results than using just one reporter [33]. Another problem in studying membrane protein is to achieve adequate expression. Some fusion recombinants do not express [40] while others can be toxic [41]. We have used a ribosomal promoter from *Burkholderia sp*. MBA4 for successful production of functional membrane protein in *E. coli*. This S12 promoter is a weak and constitutive promoter in *E. coli *and has been shown to be ideal for expression of potentially toxic membrane protein [11]. Recombinant proteins made up of Deh4p and truncated derivatives fused with PhoA and LacZα were constructed. The use of LacZα decreased the sizes of the fusion proteins. With an appropriate host that allows α-complementation [42] the LacZα will work normally. DNA fragments containing full-length and truncated *deh4p *of different lengths were amplified and cloned in-frame with the *phoA-lacZα *dual reporter genes. Thirty-six constructs were made. The truncation end points of the Deh4p were designed to end in every putative TMS or extra-membranous loops as predicted by the program SOSUI [14]. The end-points of these fusion proteins and their relative locations are illustrated in Fig. 2. *E. coli *transformants, each carrying a plasmid expressing a fusion protein (pHKU1601 plasmid series) were shown to have similar growth rates in LB (data not shown). Moreover, the production of fusion proteins was confirmed with a color indicator plate containing X-Phos (5-Bromo-4-chloro-3-indolyl phosphate) and Red-Gal™ (6-Chloro-3-indolyl- β-D-galactoside) [33] (data not shown). This suggested that the presence of the plasmids or proteins was not affecting the general physiology of the cells.

**Figure 3:**
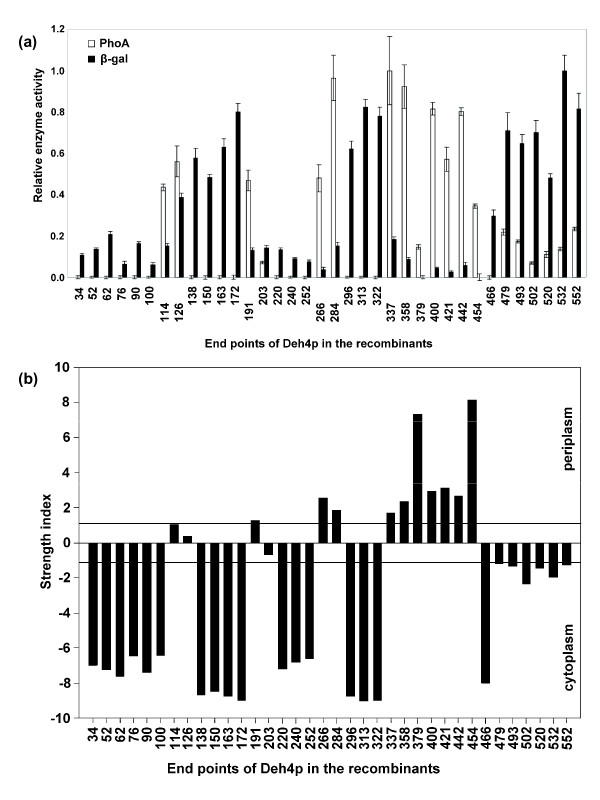
**PhoA-LacZ enzymes activities and strength indexes of cells carrying the pHKU1601 plasmid series**. (a) Relative PhoA and LacZ (β-gal) activities are presented as means ± standard error, which were obtained by linear regression through at least 20 data points obtained from 5 replicates. To normalize PhoA activities, the maximum PhoA activity recorded in the experiment (pHKU1601-337) was transformed to 1 and PhoA activities of other samples were expressed as a percentage relative to this maximum value. The same procedure was applied to normalize LacZ activities using the activity from pHKU1601-532 as the maximum. The end points of Deh4p in the recombinants are indicated. When a number is shifted downward it implies that the reporter was located in the periplasm. (b) A bar-chart showing the strength indexes of the recombinants shown in (a). When a normalized activity value was zero an arbitrary small value, 0.0001, was assigned to prevent logging a zero or undefined number in calculating the strength index. A positive value for the strength index indicates that the reporter ended in the periplasm and a negative value suggests that the reporter ended in the cytoplasm. The strength index was defined as Ln(normalized PhoA activity/normalized LacZ activity).

*E. coli *cells carrying pHKU1601 series plasmids were permeabilized with chloroform and SDS and assayed for their PhoA and LacZ activities using *p*-nitrophenyl phosphate (PNPP) and *o*-nitrophenyl galactopyranoside (ONPG) as substrates, respectively. The enzymes activities were normalized using the highest activity as one (See Additional file 1 for the data used in the analysis). The relative enzymes activities are schematically shown in Fig. 3a. There is without doubt that the expression levels among the various constructs vary from one to another. The relative strength of these two enzymes in a construct was expressed as a strength index which is the natural logarithm of the normalized activity ratio of PhoA/LacZ. The strength indexes of the constructs are shown as a bar-chart in Fig. 3b. A positive strength index indicates high PhoA activity and low LacZ activity while a negative value shows the reverse situation. Hence, when the strength indexes were sorted according to the end points of the truncated Deh4p, the presence of a TMS was implied each time the index reversed its sign. The absolute value of the index serves as a reliability indicator. If 75% of the reporters were properly localized, which is the recommended ratio for a reliable informative result [33], the normalized activity ratio for PhoA:LacZ would be 1:3 or 3:1. This ratio corresponds to a strength index of ± 1.1. A strength index higher or lower than this boundary can be considered reliable. Fig. 3b shows that the strength index changed its sign 8 times along the sequences of Deh4p and the majority of the indexes lie beyond the ± 1.1 boundary. The predicted topology and its relationship with the experimental results are illustrated in Fig. 2. Among the 36 constructs, 13 of them had junction end points in the putative periplasmic loops, twelve of them ended in the middle of the TMS and 11 of them ended in the putative cytoplasmic loops. All the 11 constructs that had the reporters in the putative cytoplasmic loops showed higher LacZ activities than PhoA activities. Among the 13 constructs that ended in the putative periplasmic loops, 11 had higher PhoA activities than LacZ activities. Two constructs, one with a fusion junction at T62 and the other at S520, had higher LacZ activities than PhoA activities. They were mapped to the first and the last putative periplasmic loop, respectively. When the reporters ended in a putative TMS, the LacZ activity was generally higher than PhoA activity regardless of the helices orientation. The only exception was observed when the reporters ended in putative TMS 4 (A126). This had higher PhoA activity than LacZ activity. The results also confirmed the presence of a long periplasmic loop stretching from residue 337 to 454. In summary, among the thirty-six fusion proteins made, only those with end-points located in putative TMS 1 and 11 and those in periplasmic loops 1 and 6 displayed contradictory results. In other words, the certainty of the presence of TMS 1 and 11 was not verified.

### Comparative analysis of Deh4p with Metabolite:H+ Symporter (MHS) family proteins

**Figure 4:**
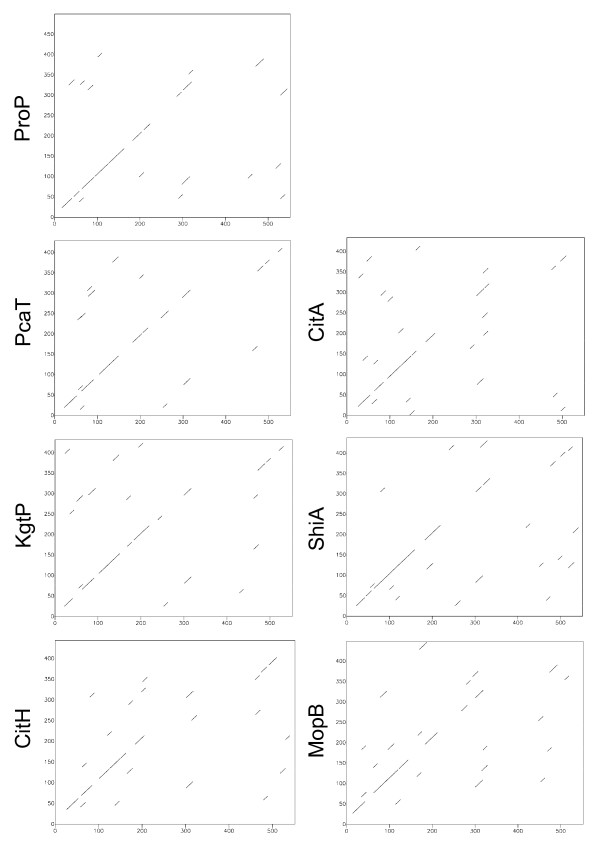
**Comparisons of Deh4p with transporter proteins of the MHS family**. The protein sequence of Deh4p (UniProt:Q7X4L6, shown as the x-axis) was compared with proteins of the MHS using dotmatcher of the EMBOSS [63]. A window size of 10, a threshold of 23 and a default matrix were used. CitH (P16482), KgtP (P0AEX3), PcaT (Q52000), ProP (P0C0L7), MopB (Q45082), ShiA (P76350) and CitA (P0A2G3) were members of TC2.A.1.6.1 to .7, respectively.

**Figure 5:**
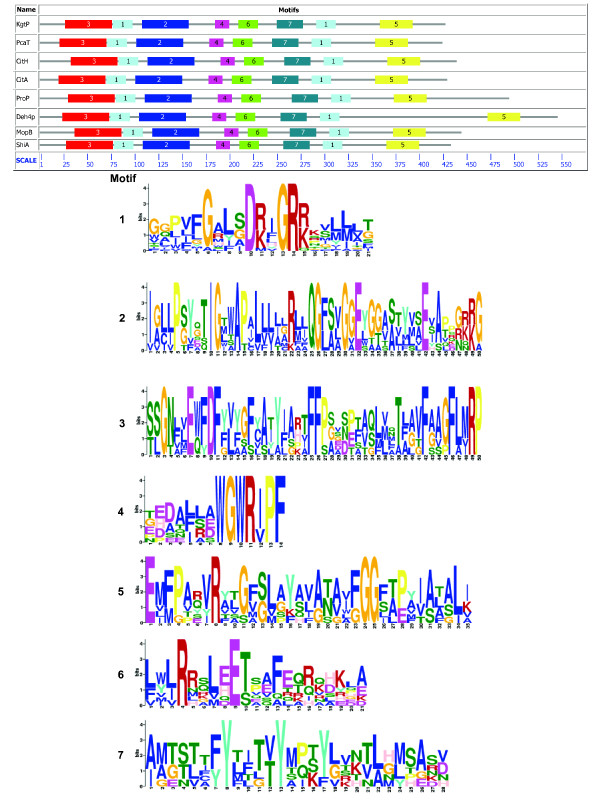
**Family-specific motifs of the MHS proteins and Deh4p**. The protein sequences of Deh4p and the MHS members (same as those used in Fig. 4) were analyzed with the motif-based analysis tools MEME [47] and MAST [48]. The top panel shows the relative locations of the conserved motifs and the lower panels show the signature sequences of the various motifs.

The current results failed to establish that Deh4p contains twelve TMS. In order to substantiate that Deh4p is a MFS protein, bioinformatic analysis was also employed. Previous comparative analyses of MFS proteins have identified specific sequence motifs [43]. A conserved motif of [RK]XGR [RK] was identified between TMS 2 and 3, and 8 and 9 of the MFS proteins. Such a motif, MIGRK (residues 86-90), was indeed identified between the predicted TMS 2 and 3 of Deh4p. A similar motif KIGRK (residues 309-313) was also found between the predicted TMS 8 and 9 of Deh4p (Fig. 2). This motif was later expanded to a conserved region of ten residues - GXXXDRXGRR - found in all 12-helix MFS proteins [44]. A consensus motif of G- [RKPATY]-L- [GAS]- [DN]- [RK]- [FY]-G-R- [RK]- [RKP]- [LIVGST]- [LIM] was also expected for all MFS proteins [23]. This motif is found between residues 81 and 93 of Deh4p. A BLASTP [45] search (accessed on 29 May, 2009) against the Transporter Classification Database [46] retrieved entries with high scores from the TC2.A.1.6 Metabolite:H^+ ^Symporter (MHS) family, subgroup of the TC2.A.1 MFS [32]. This subgroup of proteins was also predicted to have twelve TMS. When the sequence of Deh4p was compared with those of the MHS members by means of diagonal plots, homologous regions were revealed for all the comparisons (Fig. 4). Proteins CitH (UniProt: P16482), KgtP (P0AEX3), PcaT (Q52000), ProP (P0C0L7), MopB (Q45082), ShiA (P76350) and CitA (P0AA2G3) exhibited homologous regions with Deh4p especially at the N-terminal. This verified that Deh4p is a MHS family protein. Since MFS protein specific signature sequences [23] were identified in Deh4p, motif-based sequence analysis programs MEME [47] and MAST [48] were thus used to analyze Deh4p and the MHS proteins. Fig. 5 shows that there are seven motifs shared by Deh4p and all the MHS members, with motif 1 found twice in every member. The signature of each motif is illustrated in logos format [49]. The order of these motifs was also common among Deh4p and the MHS members. This verified that Deh4p is without doubt a MHS family protein and is likely to have similar structure as other MFS proteins.

## Discussion

Haloacid permease Deh4p of *Burkholderia *sp. MBA4 was classified as a member of the MFS based on its sequence similarity [10]. It was predicted to have twelve TMS. In this study dual-reporters - PhoA-LacZ - were used to study the topology of Deh4p. Thirty-six Deh4p-PhoA-LacZ constructs were made and the fusion proteins expressed in *E. coli*. Analyses of the PhoA and LacZ activities of these constructs verified that the N- and the C-termini were located in the cytoplasm. This is typical for many MFS proteins [24]. The experimentally determined topology of Deh4p was, however, slightly different from typical MFS transporters. Fusion proteins with Deh4p junctions at G52, T62 and S520 were expected to show a higher PhoA than LacZ activity. Cells expressing these fusion proteins actually exhibited higher LacZ activity. This suggested that the presence of the first and the eleventh TMS was not verified. It is possible that these helices have a low average hydrophobicity. Fig. 1 shows that this is indeed the case for TMS 1 and 11.

It can be argued that the presence of a LacZ moiety affected the translocation and correct folding of the PhoA, and thus its activity, in the periplasm. This is rather unlikely as only the LacZα fragment was used. Moreover, if this were true then the shorter the periplasmic loop the more likely that the PhoA activity will be concealed. The second predicted periplasmic loop only has a size of one residue (G114), and cells producing Deh4p_1-114_-PhoA-LacZ has a positive strength index. This indicated that the dual-reporter registered the location of the periplasmic loop accurately. Another concern arising from using enzymatic reporter assay for topology study is insufficient understanding of the details of membrane protein topogenesis. This concern is very real as current knowledge of topogenesis and membrane insertion mechanisms mainly comes from studies of eukaryotic cell organelles [50-53]. The topology of the transporter may alter if it is truncated and attached to another domain [33].

Inconclusive illustration of the presence of the TMS by the fusion reporter system has been reported. When -PhoA and -LacZ fusions were constructed near the N-terminal of the Na^+^/proline transporter PutP of *E. coli*, similar enzyme activities were detected [54]. Helix I of the *E. coli *α-ketoglutarate permease KgtP was not detected by a PhoA fusion [55]. In this case the presence of positively charged residues in other TMS was required to neutralize the negatively charged residues (E34 and D37) in helix I in order to place the segment into the membrane correctly. Similar negatively charged amino acids in Deh4p (E31 and D34) were predicted to be situated in the cytoplasm by the SOSUI program but were postulated to be part of helix I by the TOPCON program. It is possible that a similar effect was currently observed.

When the PhoA-LacZ reporter system was first developed, it was tested on the LacY protein. Eight of the LacY-PhoA-LacZα recombinants had the reporters ending in the TMS and seven of them were found to have higher PhoA than LacZ activities regardless of the orientation of the TMS [33]. This is in contrast to the present study where higher LacZ than PhoA activities were detected in the majority of the recombinants with reporters that ended in the middle of a TMS, regardless of the orientation of the TMS (Fig. 2). The inability of the method to mark the boundary of the TMS and the tendency to have higher LacZ activity suggested the risk of having TMS omitted if insufficient number of constructs were made. The use of an *E. coli *strain, TOP10, with a wildtype *phoA *gene did not affect the quantification of the PhoA activities. The background enzyme level was negligible in all our experiments. This is similar to cases where a strain, TG1, which has a wildtype *phoA *gene, was used [33,56].

The use of a fusion reporter system also failed to characterize membrane protein with atypical features. Helices E-F and P-Q of the *E. coli *ClcA protein, which has a known 3-D structure, were not detected by PhoA and green fluorescent protein fusions [40]. These helices may have formed helical hairpins [57] and inserted into the membrane at a later stage of the folding [40]. Further analysis is required to establish whether TMS 1 and 11 of Deh4p have a similar property. Further examination of hydropathy [58] and amphipathicity [59] plots by visual inspection also revealed that Deh4p may have less than twelve TMS. High amphipathicity with high hydrophobicity were also observed for the first 90 residues. This is unusual since TMS of structurally known MFS proteins LacY [26], EmrD [25], GlpT [27] and OxlT [28,29] have high hydrophobicity but not amphipathicity. These analyses suggested that Deh4p may be an atypical MFS.

Comparative analysis of Deh4p with members of TC2.A.1.6 group indicated that it shares a lot of common features with this group of MFS proteins. Not only do they have seven conserved motifs, the organization of these motifs is also similar among the different members. Motif 1, which appeared twice, is the signature region linking TMS 2 and 3, and 8 and 9 of all MFS proteins. These family-specific motifs demonstrated that Deh4p is both a MHS and MFS protein. However, residues spanning 340 to 450 of Deh4p are unique among the MHS. This region is the periplasmic loop of Deh4p. A FASTA [60] and a BLASTP [45] search of the protein database UniProt Knowledgebase (UniProtKB) using this loop sequence have identified putative MFS proteins only from the α-, β-, γ- and δ-Proteobacteria. It is likely that this loop region is specific for the transporter proteins found in Proteobacteria except the ε-Class. The role of this loop awaits further study. The presence of such a loop near the C-terminal suggested that Deh4p is not the result of simple tandem duplication and is atypical of MFS proteins. During the preparation of this manuscript Deh4p has been designated as TC2.A.1.6.8 to indicate its difference from the other MHS members.

## Conclusion

The use of PhoA-LacZα dual reports is a simple and convenient method to determine the general topology of any membrane protein. Together with bioinformatic analyses it is possible to produce a more reliable model for the protein being examined. Deh4p has been demonstrated to be an atypical MFS protein with an asymmetric organization and a long periplasmic loop. Although high-resolution structural study is ultimately required to elucidate the actual structure of Deh4p with certainty, the current data are sufficient to conclude the major structural features of Deh4p.

## Methods

### Strains and culture conditions

*E. coli *TOP10 (Invitrogen) was used for gene cloning and expression of the fusion proteins. *E. coli *cells were grown at 37°C in Luria broth (LB, 1% tryptone, 0.5% yeast extract, 0.5% NaCl) with or without 100 μg/ml ampicillin. *Burkholderia *sp. MBA4 (previously *B. cepacia*) was isolated from soil using monobromoacetate as the growth enrichment substrate [8]. MBA4 was grown at 30°C in Luria broth without NaCl.

### Construction of PhoA-LacZ reporter plasmids

DNA fragment encoding PhoA and LacZα was PCR amplified from plasmid pMA632 [33] with primers SpeI-reporter-F (5'-ACTAG TGTTC TGGAA AACCG GGCTG CTCA-3') and Reporter-stop-R (5'-GAGCT TCATT CGCCA TTCAG GCTGC GCAAC TG-3'). The amplified fragment was cloned downstream of the *lac *promoter of vector pCR2.1-TOPO by TOPO-TA cloning (Invitrogen). A plasmid with the reporters in the correct orientation was designated as pHKU1433. Ribosomal promoter S12 of MBA4 (P_*s12*_) was amplified from MBA4 total DNA with primers HindIII-S12-Fwd (5'-AAGCT TCGCA AGCCG TTGAC TTAGT TGG-3') and S12-BsiWI-Rev (5'-CGTAC GACCA GTTGG TTGAT GG-3'). The *deh4p *gene was similarly amplified with primers BsiWI-4p-Fwd (5'-CGTAC GGATG GCGAC TATTG A-3') and 4p552R-speI (5'-ACTAG TGTCC GCGTC ATAGG TAGAA GAACC CTT-3'). Both PCR products were individually cloned into pGEM-T Easy vector (Promega). The P_S*12*_-containing fragment was subsequently isolated by digesting the plasmid with *Hind*III and *Bsi*WI. The *deh4p*-bearing fragment was isolated by digesting the plasmid with *Bsi*WI and *Spe*I. These DNA fragments were mixed with *Hind*III and *Spe*I cut pHKU1433 and ligated with T4 DNA ligase. A plasmid with P_s*12*_-*deh4p *ligated upstream of *phoA-lacZ *was assembled and named as pHKU1601-552.

Truncated derivatives containing partial *deh4p *were constructed by amplifying P_*s12*_and *deh4p *from pHKU1601-552 using primer HindIII-S12-Fwd and a reverse primer 4pXYZR-speI where XYZ stands for the end point of the residue number of Deh4p. The names and sequences of the reverse primers used are shown in Table 1. The amplified fragments were cloned into pGEM-T Easy and isolated by cutting with *Hind*III and *Spe*I. These fragments were then cloned into *Hind*III and *Spe*I cut pHKU1433 to form pHKU1601-XYZ where XYZ is defined as previously. A total of 35 truncated derivatives were constructed.

**Table 1:** Reverse primers used for the construction of plasmid pHKU1601 series.

Plasmid	Primer	Sequence (5' to 3')
pHKU1601-034	4p034R-speI	ACTAG TGTCA TACCA CTCGA ATACG GTTCC CAA

pHKU1601-052	4p052R-speI	ACTAG TACCG GAGAA GAACG TTCGG CT

pHKU1601-062	4p062R-speI	ACTAG TTGTG AACAC AAACC CCGCT GCTG

pHKU1601-076	4p076R-speI	ACTAG TGCCA AAAGG ACGCA CGGCG

pHKU1601-090	4p090R-speI	ACTAG TCTTG CGTCC GATCA TGTCT CCAAG

pHKU1601-100	4p100R-speI	ACTAG TCATC AGCAG GATTG TCGCA AGAA

pHKU1601-114	4p114R-speI	ACTAG TTCCG TAACC GGGCA ACAAT CCAA

pHKU1601-126	4p126R-speI	ACTAG TAGCG ATGAA AACAA CCGGC GC

pHKU1601-138	4p138R-speI	ACTAG TCTCT CCGCC AAGCG CCAG

pHKU1601-150	4p150R-speI	ACTAG TTGCG TGTTC CGCAA CATAG GTC

pHKU1601-163	4p163R-speI	ACTAG TCTGG ATCCA TGCGG TCCAT GCG

pHKU1601-172	4p172R-speI	ACTAG TAATA AACAG GCCAA GCGTA GCCGT

pHKU1601-191	4p191R-speI	ACTAG TGGCC GCAAA TGTAT CTTCG TTAAG CAA

pHKU1601-203	4p203R-speI	ACTAG TAACG ATCGA GACAA GGAAA GGAAC G

pHKU1601-220	4p220R-speI	ACTAG TAACG GGTGA CTCGT GAAGT TGC

pHKU1601-240	4p240R-speI	ACTAG TCCCG AATGC TTCCG ATAGT GGGG

pHKU1601-252	4p252R-speI	ACTAG TTAGT GCAAG CAGGA CGATT TTCAG

pHKU1601-266	4p266R-speI	ACTAG TGCCC GTGTA CCATA CAACC GCC

pHKU1601-284	4p284R-speI	ACTAG TGCTC GTACC GTCGA CCTTA AGAGT CTG

pHKU1601-296	4p296R-speI	ACTAG TACCG ATCAG CAACG CGACA G

pHKU1601-313	4p313R-speI	ACTAG TCTTT CGCCC AATCT TGTCC GACAG

pHKU1601-322	4p322R-speI	ACTAG TAATC AGGCA GCCTG CCATG ATA

pHKU1601-337	4p337R-speI	ACTAG TGTAG TGGGC GAGAG CCTTG AAC

pHKU1601-358	4p358R-speI	ACTAG TGCTC GGATC AGCGA TCATC G

pHKU1601-379	4p379R-speI	ACTAG TTGCG ACGTC ACACG AACTC G

pHKU1601-400	4p400R-speI	ACTAG TGACA GTCCC GGCAG GGGC

pHKU1601-421	4p421R-speI	ACTAG TTTTT GCGTC CGCCG CTTTC

pHKU1601-442	4p442R-speI	ACTAG TGGCG GGGTA GCCAG CAGTC T

pHKU1601-454	4p454R-speI	ACTAG TCGAC ATCGG CCAGT TGATC AGCG

pHKU1601-466	4p466R-speI	ACTAG TGGTG ACGTA GAGCA CGAGT ATCGT CAG

pHKU1601-479	4p479R-speI	ACTAG TCATC TCCAC CAGCA TTGCT GCG

pHKU1601-493	4p493R-speI	ACTAG TATAA GGCAG CGACA TTGAG GTGTA TCG

pHKU1601-502	4p502R-speI	ACTAG TGCCG CCGAA CCAGC CATTG

pHKU1601-520	4p520R-speI	ACTAG TTGAA TAGAT GTTCC CGCGC GCTG

pHKU1601-532	4p532R-speI	ACTAG TCGCA ACGGA AGCGA TAACA ATC

pHKU1601-552	4p552R-speI	ACTAG TGTCC GCGTC ATAGG TAGAA GAACC CTT

### Assay of PhoA and LacZ activities

*E. coli *cells containing pHKU1601- series plasmid were grown in 5 ml Luria Broth with 100 μg/ml ampicillin. The cultures were incubated overnight at 37°C with shaking. One milliliter of overnight culture was saved for β-galactosidase (LacZ) assay and another milliliter for alkaline phosphatase (PhoA) assay. The protocols for PhoA and LacZ activity assay were modified from a previous report that utilized 96-well microtiter plate [61].

To determine the PhoA activity, 1 ml overnight culture was harvested, washed once in 1 ml Tris-HCl (pH 8.0) and resuspended in 1 ml assay buffer (1 M Tris-HCl, 0.1 mM ZnCl_2_, pH 8.0). The cells were permeabilized by adding 50 μl of chloroform and 50 μl of 0.1% SDS and gently vortexed for 10 sec. The mixture was incubated at 30°C for 20 minutes. After the chloroform was settled, 200 μl of the upper aqueous phase was transferred to a well of a microtiter plate. The reaction was started by the addition of 25 μl of *p*-nitrophenylphosphate solution (Sigma, N7653) and kept at 30°C. Formation of *p*-nitrophenol was measured by absorbance at 405 nm at 2 minutes' interval, followed by 10 seconds of orbital shaking that prevent cell sedimentation, for 1 hour. The cell densities of the samples were measured by absorbance at 600 nm.

Determination of the LacZ activity was also started with a 1 ml culture but this time washed with Z-buffer [34] and resuspended in 1 ml Z-buffer with 50 mM β-mercaptoethanol. The cells were then permeabilized and transferred to a microtiter plate as in the PhoA activity assay. The reaction was started by the addition of 25 μl of *o*-nitrophenyl galactopyranoside (Sigma, N1127; 4 mg/ml in Z-buffer). Formation of *o*-nitrophenol was quantified by absorbance at 420 nm in conditions similar to that of PhoA assay. The cell densities of the samples were also recorded.

To determine the relative strength of PhoA and LacZ activities, the raw rate of substrate turnover for sample *i*, *R*_*i*_, was determined by fitting a straight line along the absorbance data where a stable and maximum rate was observed. The slope of this line is *R*_*i*_. A dimensionless index, *I*, was developed for easy interpretation of data, where

The terms *R*_*i*_, _*PhoA *_and *R*_*i*_, _*LacZ *_represent *R*_*i *_for the PhoA and the LacZ assays, respectively. *D*_*i*, *LacZ *_and *D*_*i*, *PhoA *_represent the optical densities at 600 nm for sample *i *in the LacZ and the PhoA assays, respectively. The term max *(R*_*i*, *LacZ *_/*D*_*i*, *LacZ*_)_*i *= 1...*n *_represents the maximum *R*_*i*_/*D*_*i *_value recorded among *n *samples for the LacZ assays and likewise the term max *(R*_*i*, *PhoA *_/*D*_*i*, *PhoA*_)_*i *= 1...*n *_represents the highest *R*_*i*_/*D*_*i *_value registered for the PhoA assays. A natural logarithm (*Ln*) was taken for the calculated value so that a positive *I *represents a higher PhoA than LacZ activity, while a negative *I *indicates that the LacZ activity was higher. Note that *R*_*i *_must be larger than zero to avoid calculation error. If *R*_*i *_was found to be zero or negative, an arbitrary small positive value was assigned.

## Authors' contributions

YMT and MY carried out the molecular biological studies and drafted the manuscript. JSHT conceived of the study, carried out the comparative analysis, participated in the design and coordination of the study and drafted the manuscript. All authors read and approved the final manuscript.

## Supplementary Material

Additional file 1 PhoA and LacZ enzymes activities of *E. coli *cells carrying pHKU1601 series plasmids. Click here for file
