# Supplementary material for: Topological analysis of a haloacid permease of a Burkholderia sp. bacterium with a PhoA-LacZ reporter
Source: BMC Microbiol. 2009 Oct 31;9:233. doi: 10.1186/1471-2180-9-233 (PMC2777183; doi:10.1186/1471-2180-9-233)
Supplement: Additional file 1 — PhoA and LacZ enzymes activities of E. coli cells carrying pHKU1601 series plasmids. [file 1471-2180-9-233-S1.pdf]

Additional file 1. PhoA and LacZ enzymes activities of *E. coli* cells carrying pHKU1601 series plasmids.

Plasmid	Fusion[a]	PhoA activity rate[b]	LacZ activity rate[c]	Cell density PhoA[d]	Cell density LacZ[e]	Relative PhoA activity[f]	Relative LacZ activity[g]	Strength index[h]	Location[i]
pHKU1601-034	D34	0.0000	0.0064	0.1681	0.1713	0.0001 ±0.0082	0.1089 ±0.0068	-6.993	C
pHKU1601-052	G52	0.0000	0.0070	0.1454	0.1493	0.0001 ±0.0050	0.1357 ±0.0084	-7.213	C
pHKU1601-062	T62	0.0000	0.0097	0.1501	0.1373	0.0001 ±0.0060	0.2066 ±0.0143	-7.633	C
pHKU1601-076	G76	0.0000	0.0034	0.1640	0.1548	0.0001 ±0.0074	0.0641 ±0.0141	-6.464	C
pHKU1601-090	K90	0.0000	0.0078	0.1501	0.1385	0.0001 ±0.0067	0.1633 ±0.0090	-7.398	C
pHKU1601-100	M100	0.0000	0.0033	0.1803	0.1602	0.0001 ±0.0042	0.0599 ±0.0117	-6.395	C
pHKU1601-114	G114	0.0458	0.0111	0.2450	0.2109	0.4351 ±0.0167	0.1527 ±0.0114	1.047	P
pHKU1601-126	A126	0.0619	0.0335	0.2573	0.2533	0.5600 ±0.0754	0.3850 ±0.0228	0.375	P
pHKU1601-138	E138	0.0000	0.0420	0.2766	0.2123	0.0001 ±0.0065	0.5769 ±0.0466	-8.660	C
pHKU1601-150	A150	0.0000	0.0382	0.1973	0.2299	0.0001 ±0.0088	0.4840 ±0.0130	-8.485	C
pHKU1601-163	Q163	0.0000	0.0459	0.2501	0.2128	0.0001 ±0.0081	0.6284 ±0.0406	-8.746	C
pHKU1601-172	I172	0.0000	0.0723	0.3168	0.2639	0.0001 ±0.0102	0.7988 ±0.0420	-8.986	C
pHKU1601-191	A191	0.0336	0.0064	0.1672	0.1431	0.4677 ±0.0492	0.1303 ±0.0121	1.278	P
pHKU1601-203	V203	0.0063	0.0083	0.2050	0.1690	0.0712 ±0.0070	0.1428 ±0.0124	-0.697	C
pHKU1601-220	V220	0.0000	0.0069	0.1865	0.1522	0.0001 ±0.0073	0.1330 ±0.0106	-7.193	C
pHKU1601-240	G240	0.0000	0.0045	0.1698	0.1450	0.0001 ±0.0044	0.0896 ±0.0072	-6.798	C
pHKU1601-252	L252	0.0000	0.0040	0.1779	0.1578	0.0001 ±0.0057	0.0745 ±0.0091	-6.613	C
pHKU1601-266	G266	0.0371	0.0020	0.1804	0.1548	0.4785 ±0.0661	0.0384 ±0.0107	2.523	P
pHKU1601-284	S284	0.0615	0.0069	0.1484	0.1331	0.9647 ±0.1112	0.1513 ±0.0172	1.853	P
pHKU1601-296	G296	0.0000	0.0460	0.2352	0.2158	0.0001 ±0.0049	0.6213 ±0.0374	-8.734	C
pHKU1601-313	K313	0.0000	0.0378	0.1612	0.1340	0.0001 ±0.0048	0.8222 ±0.0375	-9.015	C
pHKU1601-322	I322	0.0000	0.0449	0.1883	0.1677	0.0001 ±0.0062	0.7800 ±0.0436	-8.962	C
pHKU1601-337	Y337	0.0616	0.0085	0.1435	0.1349	1.0000 ±0.1665	0.1829 ±0.0134	1.699	P
pHKU1601-358	S358	0.0637	0.0041	0.1611	0.1348	0.9216 ±0.1059	0.0884 ±0.0069	2.345	P
pHKU1601-379	A379	0.0104	0.0000	0.1646	0.1437	0.1472 ±0.0106	0.0001 ±0.0067	7.294	P
pHKU1601-400	V400	0.0506	0.0019	0.1445	0.1273	0.8150 ±0.0309	0.0435 ±0.0062	2.931	P
pHKU1601-421	K421	0.0302	0.0011	0.1233	0.1285	0.5709 ±0.0587	0.0254 ±0.0072	3.114	P
pHKU1601-442	A442	0.0473	0.0025	0.1374	0.1281	0.8011 ±0.0191	0.0572 ±0.0143	2.639	P
pHKU1601-454	S454	0.0222	0.0000	0.1501	0.1348	0.3441 ±0.0112	0.0001 ±0.0166	8.143	P
pHKU1601-466	T466	0.0000	0.0140	0.1527	0.1381	0.0001 ±0.0090	0.2952 ±0.0288	-7.990	C
pHKU1601-479	M479	0.0176	0.0423	0.1872	0.1740	0.2194 ±0.0154	0.7083 ±0.0873	-1.172	C
pHKU1601-493	Y493	0.0105	0.0306	0.1415	0.1374	0.1736 ±0.0086	0.6481 ±0.0424	-1.317	C
pHKU1601-502	G502	0.0046	0.0377	0.1568	0.1566	0.0687 ±0.0078	0.7006 ±0.0572	-2.323	C
pHKU1601-520	S520	0.0078	0.0249	0.1633	0.1512	0.1106 ±0.0141	0.4803 ±0.0209	-1.468	C
pHKU1601-532	A532	0.0104	0.0545	0.1757	0.1588	0.1381 ±0.0076	1.0000 ±0.0754	-1.980	C
pHKU1601-552	D552	0.0144	0.0336	0.1438	0.1201	0.2338 ±0.0101	0.8142 ±0.0745	-1.248	C

[a] Position of the last residue of Deh4p followed by reporter.

[b] Average PhoA activity rate was determined by the conversion of *p*-nitrophenylphosphate (PNPP) to *p*-nitrophenol. Formation of *p*-nitrophenol was measured by absorbance at 405 nm as described in Methods.

[c] Average LacZ activity rate was determined by the conversion of *o*-nitrophenylgalactoside (ONPG) to *o*-nitrophenol. Formation of *o*-nitrophenol was measured by absorbance at 420 nm as described in Methods.

[d] and [e] Average cell density used in assaying PhoA or LacZ activity was determined by absorbance at 600 nm as described in Methods.

[f] and [g] PhoA and LacZ activities are presented as mean ±S.E. which were obtained by linear regression through at least 20 data points. To normalize PhoA activities, the maximum PhoA activity recorded in the experiment (pHKU1601-337) was transformed to 1 or 100% and PhoA activities of other samples were expressed as a percentage relative to this maximum activity. The same procedure was applied to normalize LacZ activities using the activity from pHKU1601-532 as the maximum. Exception was required when a normalized activity value was zero. In this case an arbitrary small value, 0.0001, was assigned to prevent logging a zero or undefined number in calculating the strength index.

[h] and [i] A positive value for the strength index indicates that the reporter ended in the periplasm (P) and a negative value suggests that the reporter ended in the cytoplasm (C). The strength index was defined as Ln(normalized PhoA activity/normalized LacZ activity).
